# tbvar: a comprehensive genome variation resource for *Mycobacterium tuberculosis*

**DOI:** 10.1093/database/bat083

**Published:** 2014-01-09

**Authors:** Kandarp Rakeshkumar Joshi, Heena Dhiman, Vinod Scaria

**Affiliations:** ^1^CSIR Open Source Drug Discovery Unit, Anusandhan Bhawan, Delhi 110001, India; ^2^Academy of Scientific and Innovative Research (AcSIR), Anusandhan Bhawan, Delhi 110001, India; ^3^Department of Biotechnology, Delhi Technological University, Bawana Road, Delhi 110042, India and ^4^GN Ramachandran Knowledge Center for Genome Informatics, CSIR Institute of Genomics and Integrative Biology (CSIR-IGIB), Mall Road, Delhi 110007, India

## Abstract

*Mycobacterium tuberculosis*, along with closely related species, commonly known as *M. tuberculosis* complex (MTBC), causes tuberculosis in humans and other organisms. Tuberculosis is a disease with high morbidity and mortality, especially in the third world. The genetic variability between clinical isolates of MTBC has been poorly understood, although recent years have seen the re-sequencing of a large number of clinical isolates of MTBC from around the world. The availability of genomic data of multiple isolates in public domain would potentially offer a unique opportunity toward understanding the variome of the organism and the functional consequences of the variations. This nevertheless has been limited by the lack of systematic curation and analysis of data sets available in public domain. In this report, we have re-analyzed re-sequencing data sets corresponding to >450 isolates of MTBC available in public domain to create a comprehensive variome map of MTBC comprising >29 000 single nucleotide variations. Using a systematic computational pipeline, we have annotated potential functional variants and drug-resistance-associated variants from the variome. We have made available this data set as a searchable database. Apart from a user-friendly interface, the database also has a novel option to annotate variants from clinical re-sequencing data sets of MTBC. To the best of our knowledge, tbvar is the largest and most comprehensive genome variation resources for MTBC.

**Database URL:** http://genome.igib.res.in/tbvar/

## Introduction

The decade following the sequencing of *Mycobacterium tuberculosis* genome has witnessed tremendous advances in the field of genomics. These changes have been propelled by significant improvements in scale, throughput and consequent drastic reduction in the cost of genome sequencing. Present nucleotide sequencing technologies have provided a new avenue to investigate pathogens in clinical settings, while also posing new challenges in comprehending the function and biological consequence of variations. The recent years have seen sequencing of a large number of bacterial pathogens, including several strains of *M*. *tuberculosis* complex (MTBC), including those sequenced in clinical settings. Lack of a systematically curated resource for variations in the MTBC genomes has significantly compromised the systematic comparison and interpretation of genomic variations toward understanding the repertoire of variations and their functional significance.

The genomic variability in MTBC has been majorly revealed through sequencing of multiple strains. A recent report characterized the global diversity, circulating strain diversity and the evolution of MTBC through whole-genome re-sequencing (1). In a similar study, the rate of mutations in active and latent infection of MTBC has been characterized through whole-genome sequencing (2). The genome organization and evolution of the pathogen, especially in relation to its antigenic repertoire, has also been characterized through sequencing multiple strains (3). Recent re-sequencing experiments have also been able to characterize the variations between the H37Rv isolates maintained at multiple laboratories (4).

Genome sequencing of MTBC has also been recently applied extensively, unraveling the genome diversity of clinical isolates derived from a variety of geographical regions (5). In addition, genome sequencing of paleontological samples has been lately used to trace genome evolution (5), adding to the spectrum of diversity information available from whole-genome sequences of MTBC. Apart from these, a number of strains associated to distinct phenotypes including drug resistance and mechanisms of evolution of drug resistance have been studied through genome sequencing (6).

The necessity of a comprehensive database of genomic variations for MTBC and the challenges and roadblocks toward systematically assembling and annotating genomes on a common and comparable platform have been discussed in detail in the recent years (7). It has been suggested that such a database would serve as a starting point toward revealing the biological and epidemiological relevance of genomic variations in *M. tuberculosis*. Early attempts to characterize available information scattered in multiple resources and selected set of genes of clinical strains resulted in the creation of *M. tuberculosis* clinical isolates genetic polymorphism database (MTCID) (8). Similar databases like Mycobacterial Genome Divergence Database (MGDD) (9) also curate genomic variations from comparative genomics of a limited set of MTBC genomes. In addition, popular databases like Single Nucleotide Polymorphism Database (dbSNP) (10) and Tuberculosis Database (TBDB) (11) also curate genomic variations. Nevertheless, the bulk of genomic variations deciphered through whole-genome re-sequencing efforts are not available in an easily searchable and browsable format in public domain.

In this report, we describe a comprehensive resource that houses systematically analyzed re-sequencing data sets of MTBC from various laboratories in public domain. This data set encompasses >450 strains, >29 000 variations making it the most comprehensive compendium of genomic variations in MTBC till date. Using a systematic computational pipeline, we have characterized potential genomic variations with functional consequences and association with drug resistance. We also show that the resource provides a near-comprehensive repertoire of common genomic variations in the organism, making it the starting point for comparative analysis of genomic variations in MTBC. In this study, we show how a comprehensive resource like tbvar could be potentially used for clinical applications. To the best of our knowledge, tbvar is the largest and most comprehensive genome variation resources for MTBC. This resource is available for free access at http://genome.igib.res.in/tbvar.

## Datasets and methods

### Genome data sets

The available re-sequencing data sets in public domain were extensively used to compile the MTBC variome. We retrieved 37 data sets from re-sequencing projects of *M. tuberculosis* from the NCBI Sequence Read Archive (SRA). These data sets correspond to 469 isolate samples. All data sets were part of re-sequencing projects using next-generation sequencing. Only data sets in public domain and not obviously under any access embargo were considered for our analysis. The data sets and metadata are summarized in Supplementary Data S1. The H37Rv reference genome (NC_000962.2) was used as the reference for mapping the reads.

### Read mapping and variant calling

We used a popular and extensively used quality-aware reference mapping toolkit Mapping and Assembly with Quality (MAQ) (12). Base-wise mean quality of reads was deduced using FastQC tool [http://www.bioinformatics.babraham.ac.uk/projects/fastqc/]. Similar quality and coverage thresholds were used by other groups to annotate variants from short reads (13). Nevertheless, our thresholds were on the higher side to improve stringency. For each run, read lengths >15 nt, with mean Phred quality score >20, were selected for reference alignment, whereas rest of the data sets were discarded. The consensus mapping quality was also used to weed out low-quality assemblies. Further, all data sets that did not match quality criteria of mean Phred score cutoff of 20 across the genome were dropped from further analysis. The resulting data set comprised 469 unique samples. The variants were called using the MAQ variant caller using parameters described in the Supplementary Data S2.

### Variant comparison

We retrieved variants from dbSNP and *Mycobacterium tuberculosi*s Clinical Isolates Genetic Polymorphism Database (MTCID) for comparisons. MTCID is a repository providing access to the genetic polymorphisms from clinical isolates and also information on their strains and associated spoligotypes (8), while dbSNP archives genomic variants of organisms. Briefly, the database had 3885 and 263 variants annotated, respectively, on the *M. tuberculosis* H37Rv genome. We also compared our repertoire of genomic variations with the collection released by TBDB on their site. This collection comprises variations obtained from diversity sequencing of 25 odd *M. tuberculosis* isolates obtained from global population. This data set comprises 5995 variations in all. Additionally, the data sets corresponding to drug-resistance traits were retrieved from TB Drug resistance mutation database (TBDreamDB) (14). This data set comprised >1100 variants corresponding to ∼40 genes for nine antibiotics. This data set was used to annotate drug-resistant variants in our repository. The comparison of the variants generated from re-sequencing data sets was compared with the known variants reported using custom scripts. A comparison between variations in tbvar and *Mycobacterium canetti*, another pathogen from *Mycobacterium* genus, was also made. The variations were derived from tbdb.org database.

### Variant annotation

The variants were annotated using the popular variant annotation toolkit ANNOVAR (15). Briefly, the gene coordinates were formatted using bespoke scripts and annotated using ANNOVAR. Variants were annotated for a number of features like gene loci (genic, intergenic), effect (synonymous, non-synonymous, stop gain/loss), etc.

### Functional analyses of variations

The genic non-synonymous variants were analyzed further using popular toolkit Sorting Intolerant from Tolerant (SIFT) (16) for potential functional consequences. Briefly, the annotations from ANNOVAR were reformatted using custom scripts and used for analysis using SIFT. The genes list and genomic coordinates for the genes were derived from the [microbes.ucsc.edu], and the nucleotide sequences were derived from the reference genome based on these coordinates.

### Mapping of variants to regulatory regions

The recent availability of chromatin immunoprecipitation (ChIP)-seq data sets for *M. tuberculosis* was extensively used for the mapping of variants. Peak data set for 50 transcription factor ChIP-Seq experiments was retrieved from the Broad Institute repository. (http://genome.tbdb.org/annotation/genome/tbdb/RegulatoryNetwork.html). The respective positions of the peaks were downloaded, and variants were matched to the respective loci using custom scripts.

### Database construction

**Figure 1.**
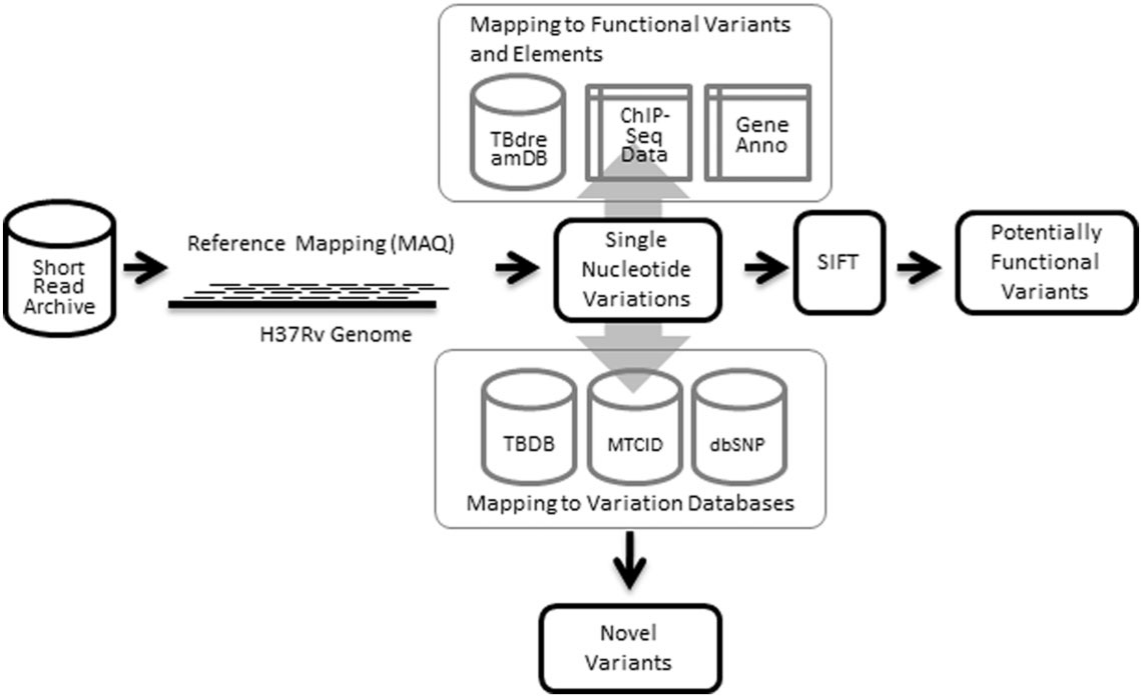
Workflow illustrating data sets and methodologies applied in building tbvar.

The database was constructed using MySQL, an open source relational database system. Interfaces were coded in Perl-CGI and HTML/CSS, and the server was hosted using Apache HTTP server. The summary of the data sets and methodology used in creating the resource is summarized as Figure 1.

### Evaluation of genomic variations saturation point from large-scale sequencing

We analyzed the comprehensiveness of our database by arguing that the novel variants discovered by adding a subset of genomes would saturate at some point if the database was near-comprehensive. To quantify the comprehensiveness of the database, we randomly chose strains from our data to quantify novel variations throughout our data set. This process was performed iteratively for 1000 iterations in randomly selected groups of five strains per group. Selection of strains in each iteration was independent of its nature or origin. A decline in the number of novel variations over iterations would signify the comprehensiveness of the variants repertoire.

### annoTB

We also developed an interface to enable researchers, characterize and annotate variations from re-sequencing data sets. The tool takes as input the variations in a pre-defined format and annotates each variation in the set for different categories as described in tbvar. One of the potential applications of such an interface is to be able to annotate data set from clinical isolates. We performed proof-of-principle analysis of a recently sequenced clinical isolate of *M. tuberculosis* OSDD271 (17) isolated from India. The variations called from this strain against H37Rv reference genome were provided to annoTB using the interface, and summary report provided by the analysis was retrieved.

## Results

### Database statistics

**Figure 2.**
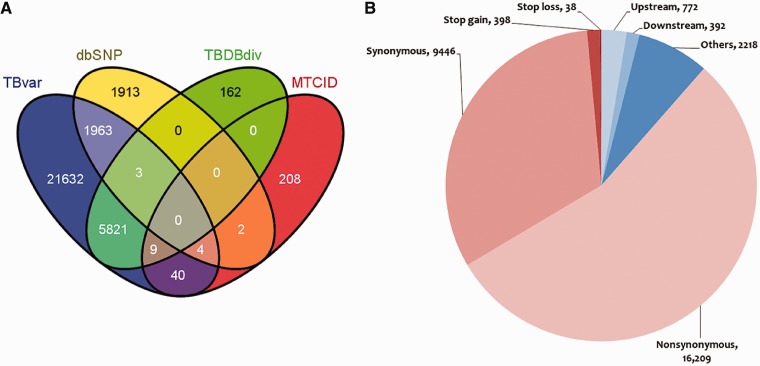
Comparison of variations with other resources and their mapping to different regions of the genome. (**A**) Comparison of the variations in *M. tuberculosis* with respect to other variation resources. (**B**) Graphical representation showing distribution of SNPs in various loci of the *M. tuberculosis* reference genome.

The database hosts >29 472 unique single nucleotide variants from 469 unique sequenced strains of *M. tuberculosis* (Supplementary Data S4). Of the total, 7824 variants could be mapped to other known variations in *M. tuberculosis* retrieved from dbSNP, MTCID and TBDB, suggesting that 21 632 variants are novel and reported for the first time in this report. The overlap of variations between each of the resources is summarized in Figure 2A. The frequency distribution of the variants is summarized in Supplementary Data S3. A sub-data set of our main data was analyzed previously (13), where consensus variation calls were made from two different tools (MAQ and BWA). However, we used more stringent variation calling criteria than the above study. Of the total single nucleotide variations (SNVs), 26 083 variations were genic, 16 209 were found to be non-synonymous SNVs (nsSNV) and 5394 were found to be deleterious in 2407 genes as predicted by SIFT, while 9446 variations were sense SNV (sSNV), and the number of variations conferring stop codon mutation to the gene was 398. The deleterious variation prediction here is considered to alter the structure of protein rather than the protein becoming non-functional. Similarly, tolerated variation prediction by SIFT is considered here to be not affecting the structure of the protein rather than the function of the protein. A total of 38 mutations led to loss of a stop codon within gene. A total of 7873 variations mapped to regulatory regions annotated as per ChIP-seq data set obtained from (http://genome.tbdb.org/annotation/genome/tbdb/RegulatoryNetwork.html). A comprehensive representation of the data is shown in Figure 2B.

The comparison of variations between tbvar and variations from *M. Canetti* showed significantly low concordance in comparison with H37Rv. The comparison of variations has been summarized in Supplementary Data S5.

### Genomic variations tend to saturation

**Figure 3.**
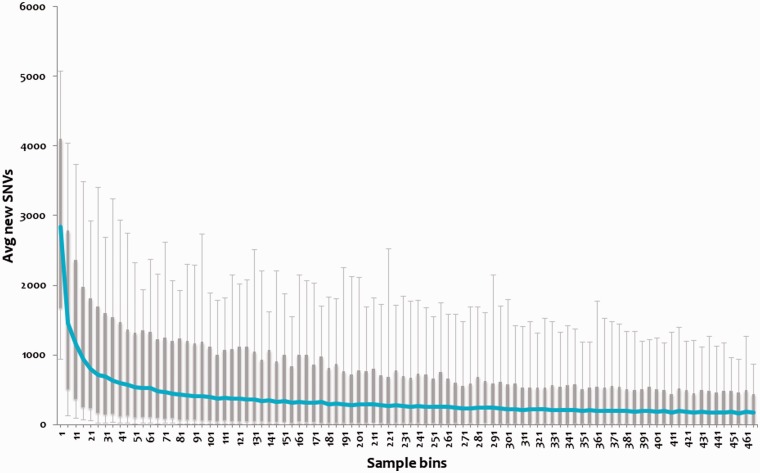
Variations plotted across subset of the genomes. Each bin of five samples was randomly chosen for 1000 iterations, and novel SNVs were identified. This number for each bin was averaged, and a box plot was plotted. The 95 percentile and 5 percentile form upper and lower boxes, while upper and lower error bars indicate maximum and minimum, respectively. The blue line passing through the boxes indicates average per bin of samples.

We analyzed the repertoire of genomic variations encoded by the 469 (samples) strains of MTBC to evaluate whether this forms a near comprehensive set of genetic variations encoded by the MTBC. We evaluated the percentage of the total repertoire contributed by randomly picked sets of genomes, and showed that the variations tend to saturate at ∼195 genomes as shown in Figure 3. Therefore, we argue that tbvar encompasses majority of the common variants encoded by the pathogen, providing a comprehensive resource and starting point toward understanding the pathogen diversity and evaluation of variations for therapeutic and drug discovery applications.

### Database features and navigation

**Figure 4.**
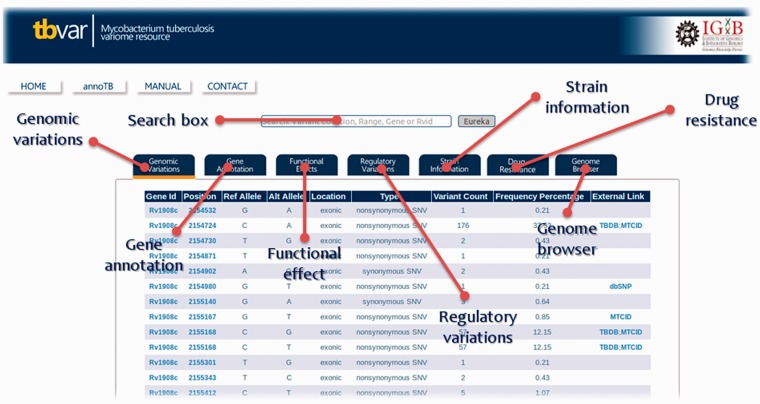
Screenshot showing result table and information or each section of database and the respective tabs for each of the sections.

The database interface has been designed to be user-friendly graphic user interface. The homepage directs the user to different search and browse options. A detailed Help Manual is also provided to aid the user on the search and navigation options. The search option has been designed to be simple and intuitive, where a user can search the database using a gene ID or a set of genomic positions and is immediately presented with a list of variants, which satisfy the condition. The search supports gene names, Tuberculist RvIDs and GenBank IDs. The ‘Eureka’ button returns results for the input search term in various tabs. Information about each tab can be obtained by hovering the mouse over the link. Each of the section displayed in the form of tab is highlighted in Figure 4. The interface also allows the user to sift through this list by using a set of tabs to filter out synonymous/non-synonymous, deleterious or variations in regulatory regions for example (Supplementary Data S6).

The homepage also features a browser interface that allows users to quickly browse for relevant information quickly. Presently, the interface supports browse options for the following features: synonymous variations, regulatory variations, deleterious variations and drug resistance variations. The search results interface also is hyperlinked so as to provide the user to dynamic compilations of information, pertaining, for example, a strain, a gene or a set of properties, say drug resistance.

To aid the user on the genomic location of the variants and the neighboring genomic context, the resource also features a browser tab displaying the variations on a genome browser interface exported from TBrowse (18).

The resource has also been interlinked to various other primary resources for gene information and sources of raw data. This includes Tuberculist and TBDB for gene information, Uniprot for protein annotations and NCBI (SRA) for raw data sets and TBrowse for genome-centered annotations. The variants in the database are already available as a track shared in TBrowse.

In addition to the web interface, variation data sets and annotations have also been made available for download to aid computational biologists with a ready set of formatted data sets for analyses.

### Application of tbvar

One of the potential applications of a comprehensive resource like tbvar would be for annotation of variants derived from re-sequencing of isolates, especially in clinical settings. tbvar provides an annotation feature ‘annoTB’, where users can input custom variant list from re-sequencing data, and annotate them for potential drug-resistant variations, retrieve allele frequencies of variations, genomic variations in regulatory regions and also retrieve information on other strains that have the same variation. In the present report, we use this feature as a proof of concept to annotate variations from a clinical isolate.

As a proof-of-principle, we have annotated the genome of a recently sequenced Multidrug resistant (MDR) strain of *M. tuberculosis* OSDD271 (17). The data were reference aligned as described in the ‘Materials and Methods’ section. The annoTB tool generated a report, which annotated the valid variations from this strain. Table 1 shows the report generated by annoTB for OSDD271 strain. As expected, this strain shows multiple variations conferring drug resistance. It also harbors 150 novel variations. In addition to annotating the strains, users have an option to contribute their novel variations to the database by entering necessary details for submission on a submit page. The submitted variations will be integrated into the database manually after curation on a regular basis. The tool is summarized in Supplementary Data S7.

**Table 1.** ‘annoTB’ variants annotation report

Total variants	Known variants	Novel variants	Drug-resistant variants	Genic variants
1454	1304	150	4^a^	1144

^a^Total drug-resistant variants present in the OSDD271 strain. The resistant variants are against ethambutol (3) and fluoroquinolone (1) antibiotics.

### Summary and future perspective

In summary, tbvar provides a much needed compendium of genomic variants and their annotations for MTBC and provides the first step toward accelerating genotype–phenotype correlations in the closely related pathogens. The database provides a user-friendly interface, closely integrated and interlinked with other major resources in the field. The tool also provides interface for annotation of known variants and identification of novel variants obtained from genome sequencing data sets and could potentially lead to application in clinical settings. We foresee drastic improvements in the compendium of genomic variants, with more genome scale data being available in public domain and without embargoes and access restrictions. We would also improve on the variant annotations with availability of genome-scale epigenetic data sets from ongoing projects, consistently improving the functional annotation of variations.

## Supplementary data

Supplementary data are available at *Database* Online.
